# Variants of the arachidonate 5-lipoxygenase-activating protein (*ALOX5AP*) gene and risk of ischemic stroke in Han Chinese of eastern China^☆^

**DOI:** 10.1016/S1674-8301(11)60043-2

**Published:** 2011-09

**Authors:** Gannan Wang, Yao Wang, Hao Sun, Weijuan Cao, Jing Zhang, Hang Xiao, Jinsong Zhang

**Affiliations:** a Emergency Center, the First Affiliated Hospital of Nanjing Medical University, Nanjing, Jiangsu 210029, China;; b Laboratory of Neurotoxicology, School of Public Health, Nanjing Medical University, Nanjing, Jiangsu 210029, China.

**Keywords:** arachidonate 5-lipoxygenase-activating protein, ischemic stroke, variants, leukotriene B4, risk factors

## Abstract

Variants of the arachidonate 5-lipoxygenase-activating protein (*ALOX5AP*) gene have been suggested to play an important role in the pathogenesis of atherosclerosis and ischemic stroke. This study was aimed to explore the association of *ALOX5AP* variants with ischemic stroke risk in Han Chinese of eastern China. A total of 690 ischemic stroke cases and 767 controls were recruited. The subjects were further subtyped according to the Trial of Org 10172 in Acute Stroke Treatment (TOAST) criteria. On the basis of that, two polymorphisms of the *ALOX5AP* gene (rs10507391 and rs12429692) were determined by TaqMan genotyping assay. In addition, plasma leukotriene B4 (LTB4) levels were analyzed in these subjects. There was no evidence of association between the two variants of *ALOX5AP* and the risk of ischemic stroke or its TOAST-subtypes. Haplotype analysis and stratification analysis according to sex, age, body mass index, hypertension, and diabetes also showed negative association. Analysis of LTB4 levels in a subset of cases and controls revealed that LTB4 levels were significantly higher in ischemic stroke cases than in the controls (70.06±14.75 ng/L *vs* 57.34±10.93 ng/L; *P* = 0.000) and carriers of the T allele of the rs10507391 variant were associated with higher plasma LTB4 levels (*P* = 0.000). The present study suggests there is no association of the two polymorphisms in the *ALOX5AP* gene with ischemic stroke risk in Han Chinese of eastern China.

## INTRODUCTION

Stroke is a common neurological disease and one of the leading causes of severe disability and death in China[1]. The majority of strokes are of ischemic origin with an atherothrombotic trigger, accounting for 80% of all strokes[2]. Ischemic stroke is a complex multifactorial and polygenic disorder that is thought to result from interactions between an individual's genetic background and various environmental factors. Atherothrombosis is considered to be the main cause[3]. A previous study had established age, sex, obesity, smoking, hypertension, diabetes, and dyslipidemia as reliable stroke risk predictors[4]. However, these conventional risk factors do not fully account for the overall risk of stroke. Evidences from twins, family, and animal studies have consistently suggested a genetic contribution to the risk of ischemic stroke[5].

Given that both systemic and local inflammatory processes are implicated in the etiology of ischemic cerebrovascular disease and in the pathophysiology of cerebral ischemia[6], polymorphisms of proinflammatory genes may contribute to the increase of susceptibility to ischemic stroke. A previous study highlighted the implication of single nucleotide polymorphisms (SNPs) and at-risk haplotypes in the arachidonate 5-lipoxygenase-activating protein (*ALOX5AP*) gene, conferring an increased risk of suffering from stroke in the Icelandic population through genome-wide linkage scan[7].

The human *ALOX5AP* gene is located on chromosome 13q12-13, including the 5 known exons and introns. It encodes 5-lipoxygenase-activating protein (FLAP or ALOX5AP), which is a regulator of the leukotriene (LT) biosynthetic pathway[7]. LT biosynthetic pathway comprises a family of arachidonic acid metabolites, which play an important role in the pathogenesis of atherosclerosis and inflammatory diseases, including ischemic stroke. In this biosynthetic pathway, unesterified arachidonic acid is converted to leukotriene A4 (LTA4) by the action of 5-lipoxygenase (5-LO) and its activating protein ALOX5AP. The unstable epoxide LTA4 is further metabolized to leukotriene B4 (LTB4) or leukotriene C4 (LTC4) by LTA4 hydrolase (LTA4H) and LTC4 synthase (LTC4S), respectively. LTB4 and LTC4 are moved out of the cell and can exert their biologic influence through specific receptors in inflammatory cells[8]. Functional polymorphisms of LT-related genes (such as *ALOX5AP*) may thereby enhance the susceptibility to stroke. Genetic effects in the LT biosynthetic pathway could be an important contributor to the development of atherosclerosis and to an increasing risk of ischemic stroke through the formation of the proinflammatory LTB4 and/or through an increase in vascular permeability caused by cysteinyl leukotrienes (LTC4 and its metabolites LTD4, LTE4)[9].

In an attempt to investigate the contribution of genetic variations in the *ALOX5AP* gene to ischemic stroke in a Chinese Han population of eastern China, a case-control association study was carried out to clarify the involvement of *ALOX5AP* genetic polymorphisms as risk factors for the pathogenesis of ischemic stroke and its subtypes.

## MATERIALS AND METHODS

### Study subjects

A total of 690 unrelated patients with a clinical diagnosis of ischemic stroke (cases) were recruited from the First Affiliated Hospital of Nanjing Medical University (Nanjing) between January 2009 and December 2010. All subjects were genetically-unrelated ethnic Han Chinese from Jiangsu Province and surrounding regions in eastern China. Stroke was defined by the presence of a new focal neurological deficit, with an acute onset and with symptoms and signs persisting for more than 24 h[10]. Ischemic stroke was confirmed in all patients by computed tomography (CT) and/or magnetic resonance imaging (MRI) as well as ancillary diagnostic investigations including duplex ultrasonography of the carotid and vertebral arteries, echocardiography, MR-angiography, CT-angiography and standardized blood tests.

Ischemic stroke cases were classified into four major subtypes according to the Trial of Org 10172 in Acute Stroke Treatment (TOAST) classification[11] by a physician reviewing original imaging and clinical reports. The TOAST subtypes include: 1) large-artery atherosclerosis (LAA); 2) small-artery occlusion (SAO), i.e. lacunar infarction; 3) cardioembolism (CE), and 4) others (including other etiologies, unknown causes, and multiple potential causes). The control group consisted of 767 unrelated individuals (controls) who were recruited simultaneously from the same geographical area as the cases. The controls had no clinical evidence of neurological diseases and were matched by age, sex, and ethnic origin. The controls included inpatients with minor illnesses (51.2%) and people undergoing annual medical examination (48.8%), free of neurovascular and cardiovascular history or family history of stroke, ascertained by direct interview before recruitment. Patients with a clinically known inflammatory, autoimmune or malignant disease were excluded from the study.

Information on demographic characteristics and other risk factors of the cases and controls was collected by investigators using the same structured questionnaire involving body mass index (BMI), smoking, alcohol consumption, family history, and history of hypertension, and diabetes. Smoking was defined as having smoked at least one cigarette per day for one year or more. Former smokers with more than five years of smoke cessation were not included[12]. Alcohol drinking was defined as at least one alcoholic drink in a week, alcohol consumption ≥50 mL and continuing for more than three months. Hypertension was defined as a systolic blood pressure ≥=140 mmHg and/or diastolic blood pressure ≥90 mmHg based on the average of the two blood pressure measurements, or a patient's self-reported history of hypertension or the use of antihypertensive drugs. Diabetes was diagnosed if fasting plasma glucose (FPG) ≥7.0 mmol/L or plasma glucose ≥11.1 mmol/L at 2 h after a 75 g oral glucose challenge or random plasma glucose≥11.1 mmol/L or the patient was on anti-diabetic medications[13].

The study protocol was reviewed and approved by the Institutional Review Boards for Human Studies of Nanjing Medical University. Informed consent was obtained from all participants.

### Clinical laboratory measurements

Whole venous blood samples were collected after at least 12 h of fasting. Fasting plasma glucose, total plasma cholesterol (TC), triglyceride (TG), high-density lipoprotein cholesterol (HDL-C), low-density lipoprotein cholesterol (LDL-C), uric acid (UA), and lipoprotein(a) [Lp(a)] were measured using an automatic biochemical analyzer (AU5400, Olympus, Tokyo, Japan).

### LTB4 production analysis

The enzyme-linked immunosorbent assay (ELISA) was performed to quantify LTB4 concentration in plasma of 45 controls and 33 ischemic stroke cases, using a R&D human LTB4 ELISA Kit protocol. One in twenty samples was analyzed in replicate to assess analytical precision.

### SNP selection and genotyping

Genomic DNA was extracted from peripheral white blood cells using the phenol/chloroform method. DNA samples were stored at -20°C. According to the information from the NCBI SNP database and HapMap database, SNPs which had a minor allele frequency (MAF) > 0.05 and those with previously reported significant associations were chosen[14]. Therefore, two SNPs (rs10507391 and rs12429692) were selected in the human *ALOX5AP* gene to determine the association between patients with ischemic stroke and controls in a Chinese Han population of eastern China. Characteristics of the investigated two SNPs are shown in ***Table 1***.

**Table 1:** Characteristics of the investigated SNPs

SNP ID	rs number	Position in chromosome	Groups			HWE *P* value in controls
			Database^a^	Cases	Controls	
1	rs10507391	31312096	0.411	0.378	0.362	0.950
2	rs12429692	31312178	0.333	0.341	0.312	0.710

MAF: minor allele frequency; HWE: Hardy-Weinberg equilibrium. ^a^ MAF for Chinese in HapMap.

The selected SNP genotypes were determined in all 1,457 subjects by the TaqMan system. PCR was conducted in ABI 9700 thermocyclers (Applied Biosystems, Inc., Foster City, CA, USA). The allelic discrimination and scoring of the genotypes were performed using the ABI 7900 HT Sequence Detection System (SDS). The ABI assay-by-design protocol was used. The probes and primers are provided in ***Table 2***. In each 384-well plate, two reference samples and two negative controls were included for quality control. Ten percent of the samples were randomly selected to perform repeated assays, and greater than 95% call rates occurred to both SNPs.

### Statistical analysis

EpiData 3.0 was used to establish the database. Deviation from the Hardy-Weinberg equilibrium (HWE) was tested by comparing the observed and expected genotype frequencies of the controls using the χ^2^ test. All continuous variables were expressed as mean±SD and compared using the unpaired Student's *t* test, unless otherwise indicated. Categorical variables were assessed by the χ^2^ test or Fisher's exact test. The haplotype frequencies were estimated with the program PHASE version 2.1, which used a Bayesian method[15]. Genotypes were assessed according to codominant, dominant, and recessive genetic models. Each genetic model was comprised of two groups: heterozygotes and variant homozygotes, respectively, *vs* wild-type homozygotes for the codominant model; the combined group of variant homozygotes and heterozygotes *vs* wild-type homozygotes for the dominant model; variant homozygotes *vs* the combined group of wild-type homozygotes and heterozygotes for the recessive model. The χ^2^ test or Fisher's exact test, as appropriate, was used to compare the distribution of genotypes and alleles of SNPs, as well as haplotypes between the cases and controls. The multivariate logistic regression model was performed to exclude the effects of the possible confounding factors (including sex, age, BMI, hypertension, and diabetes) on the association between genetic variants and ischemic stroke. The odds ratios (*ORs*) and 95% confidence intervals (95%*CIs*) for the effects of genotypes on ischemic stroke risk were uncorrected for confounding variables in the χ^2^ test and unadjusted Logistic regression, and were corrected for covariates in adjusted regression models.

Plasma LTB4 levels of the different genotypes in the cases and controls were compared using t test or ANOVA with *P* < 0.05 considered as statistically significant.

All the statistical tests were performed using SPSS version 16.0 (SPSS Inc., Chicago, USA). A two-tailed *P* value < 0.05 was considered significant.

**Table 2:** TaqMan probes and primers for genotyping *ALOX5AP* SNPs

SNPs	Probe (5′-3′)	Primer (5′-3′)
rs10507391	P-T: FAM-TGCAATTCTATTTAACCTC-MGB	F: TCACAAGATCCAGATGTATGTCCAA
	P-A: HEX-TGCAATTCTAATTAACCTC-MGB	R: CTTAAGGTAGGTCTATGGTTGCAACA
rs12429692	P-A: FAM-CTTTCTTCTTCCTCATACC-MGB	F: TTGCAACCATAGACCTACCTTACAGA
	P-T: HEX-TTCTTCTTCCTCATTCC-MGB	R: CAGGAAATGTTCAGAATGGCATT

The nucleotides of polymorphisms are underlined.

## RESULTS

### Clinical characteristics of subjects

The demographic characteristics of the controls and ischemic stroke cases are presented in ***Table 3***. The mean age was 67.87±9.52 years for the cases and 67.54±9.46 years for the controls; 59.7% cases and 54.1% controls were male. As expected, compared with the control group, the ischemic stroke group had a greater prevalence of the conventional risk factors including male, history of hypertension and diabetes, as well as significantly higher BMI, systolic and diastolic blood pressure, FPG, TC, TG, LDL-C, UA and Lp(a), and lower HDL-C. However, there were no significant differences in smoking and alcohol drinking between the two groups. In addition, varied TOAST-subtypes showed different risk factors.

**Table 3:** Demographic and clinical characteristics of the study population

Characteristics	Controls (*n* =767)	IS TOAST-subtypes				
		Total (*n* =690)	LAA (*n* =192)	SAO (*n* =355)	CE (*n* =71)	Others (*n* =72)
Age (y)	67.54±9.46	67.87±9.52	66.24±10.37	68.27±9.08	71.01±7.93^b^	67.15±9.90
Male (%)	54.1	59.7^a^	64.6^b^	62.6	63.4	45.8
BMI (kg/m^2^)	23.30±2.37	24.20±3.21^b^	24.67±3.44^b^	24.09±3.03^b^	23.91±3.38	23.78±3.23
Smoking (%)	25.2	24.9	29.2	23.7	22.5	22.2
Alcohol drinking (%)	18.5	17.5	21.4	15.5	21.1	13.9
Hypertension (%)	27.5	77.1^b^	77.1^b^	78.9^b^	78.9^b^	66.7^b^
Diabetes (%)	12.4	34.1^b^	39.1^b^	34.4^b^	26.8^b^	26.4^b^
Systolic BP (mmHg)	125.25±17.46	144.09±22.17^b^	149.44±23.15^b^	143.08±20.78^b^	140.82±26.09^b^	138.04±19.36^b^
Diastolic BP (mmHg)	79.12±30.09	83.69±11.80^b^	85.04±11.60^b^	83.59±11.34^b^	82.08±13.93	82.17±12.12
FPG (mmol/L)	5.95±2.44	6.40±2.61^b^	6.65±2.75^b^	6.37±2.54^b^	6.32±2.95	5.98±2.17
TC (mmol/L)	4.45±1.20	4.67±1.17^b^	4.66±1.13^a^	4.75±1.16^b^	4.41±1.04	4.54±1.37
TG (mmol/L)	1.37±0.95	1.67±1.19^b^	1.70±1.09^b^	1.66±1.28^b^	1.73±1.27^a^	1.60±0.88
HDL-C (mmol/L)	1.27±0.35	1.15±0.34^b^	1.13±0.31^b^	1.19±0.34^b^	1.07±0.34^b^	1.12±0.36^b^
LDL-C (mmol/L)	2.52±0.78	2.79±0.84^b^	2.84±0.83^b^	2.81±0.83^b^	2.59±0.88	2.72±0.92^a^
UA (µmol/L)	279.88±98.22	313.23±107.59^b^	315.83±110.81^b^	313.34±106.16^b^	323.08±107.91^b^	296.09±105.89
Lp(a) (mg/L)^c^	114.00±231.00	166.00±256.25^b^	162.00±222.50^b^	157.00±298.00^b^	134.00±212.00	221.00±255.25^b^

Compared with controls, ^a^*P* < 0.05, ^b^*P* < 0.01. Age, body mass index (BMI), systolic blood pressure (BP), diastolic BP, fasting plasma glucose (FPG), total cholesterol (TC), triglyceride (TG), high-density lipoprotein cholesterol (HDL-C), low-density lipoprotein cholesterol (LDL-C), and uric acid (UA) are given as mean±SD. ^c^ Lipoprotein(a)[Lp(a)] is given as median±interquartile range, and compared using Mann-Whitney test. IS: ischemic stroke; TOAST: trial of org 10,172 in acute stroke treatment; LAA: large-artery atherosclerosis; SAO: small-artery occlusion; CE: cardioembolism.

### Association between the *ALOX5AP* polymorphisms and ischemic stroke

The genotype distributions of the two SNPs in controls were consistent with the Hardy-Weinberg equilibrium (*P* > 0.05). There were no significant differences in the genotypic distributions and allelic frequencies of rs10507391 and rs12429692 between the controls and the ischemic stroke cases or its subtypes (***Table 4***). Furthermore, as shown in ***Table 5***, no evidence of association with ischemic stroke and its subtypes (data not shown) was found by using different genetic models for both SNPs. When stratification analysis was performed according to sex, age, BMI, hypertension, and diabetes, there was also no significant association of the two SNPs with ischemic stroke risk (***Table 5***). In addition, because of the two SNPs (rs10507391 and rs12429692) had shown strong linkage disequilibrium (LD) in the same block region of *ALOX5AP* (pair-wise *R*^2^ > 0.8) defined by the program Haploview version 4.2, we carried out haplotype analysis. However, compared with controls, no significant association was found with the risk of ischemic stroke (***Table 7***) and its TOAST-subtypes (data not shown) in terms of the haplotype frequencies.

**Table 4:** Genotypic distributions and allelic frequencies of rs10507391 and rs12429692

	Controls (*n* =767)	IS TOAST-subtypes				
		Total (*n* =690)	LAA (*n* =192)	SAO (*n* =355)	CE (*n* =71)	Others (*n* =72)
rs10507391 A>T						
AA *(n,* %)	100 (13.0)	97 (14.1)	22 (11.5)	56 (15.8)	8 (11.3)	11 (15.3)
AT (*n*, %)	355 (46.3)	327 (47.4)	95 (49.5)	158 (44.5)	37 (52.1)	37 (51.4)
TT (*n*, %)	312 (40.7)	266 (38.6)	75 (39.1)	141 (39.7)	26 (36.6)	24 (33.3)
* P* value^a^		0.674	0.693	0.465	0.639	0.472
A Allele (%)	36.2	37.8	36.2	38.0	37.3	41.0
* P* value^a^		0.379	0.995	0.398	0.786	0.254
rs12429692 A>T						
AA (*n*, %)	365 (47.6)	299 (43.3)	83 (43.2)	157 (44.2)	32 (45.1)	27 (37.5)
AT (*n*, %)	325 (42.4)	311 (45.1)	90 (46.9)	154 (43.4)	32 (45.1)	35 (48.6)
TT (*n*, %)	77 (10.0)	80 (11.6)	19 (9.9)	44 (12.4)	7 (9.9)	10 (13.9)
*P* value^a^		0.239	0.510	0.388	0.905	0.226
T Allele (%)	31.2	34.1	33.3	34.1	32.4	38.2
*P* value^a^		0.095	0.427	0.177	0.774	0.086

^a^ χ^2^ tests, controls *vs* ischemic stroke and its TOAST subtypes. IS: ischemic stroke; TOAST: trial of org 10,172 in acute stroke treatment; LAA: large-artery atherosclerosis; SAO: small-artery occlusion; CE: cardioembolism.

**Table 5:** Detailed association results of the SNPs between controls and ischemic stroke

Genetic model		Controls [*n*(%)]	Cases [*n* (%)]	Unadjusted		Adjusted	
				*OR* (95% *CI*)	*P*	*OR* (95% *CI*)	*P*
rs10507391							
Codominant	AA	100 (13.0)	97 (14.1)	Ref.	—	Ref	—
	AT	355 (46.3)	327 (47.4)	0.95 (0.69,1.30)	0.749	0.82 (0.57, 1.19)	0.297
	TT	312 (40.7)	266 (38.6)	0.88 (0.64,1.22)	0.434	0.85 (0.58, 1.24)	0.398
Dominant	AA	100 (13.0)	97 (14.1)	Ref.	—	Ref	—
	AT, TT	667 (87.0)	593 (85.9)	0.92 (0.68,1.24)	0.570	0.83 (0.59, 1.18)	0.304
Recessive	AA, AT	455 (59.3)	424 (61.4)	Ref.	—	Ref	—
	TT	312 (40.7)	266 (38.6)	0.92 (0.74,1.13)	0.407	0.97 (0.76, 1.24)	0.814
rs12429692							
Codominant	AA	365 (47.6)	299 (43.3)	Ref	—	Ref	—
	AT	325 (42.4)	311 (45.1)	1.17 (0.94,1.45)	0.162	1.14 (0.88, 1.47)	0.319
	TT	77 (10.0)	80 (11.6)	1.27 (0.90,1.80)	0.180	1.36 (0.91, 2.05)	0.135
Dominant	AA	365 (47.6)	299 (43.3)	Ref	—	Ref	—
	AT, TT	402 (52.4)	391 (56.7)	1.19 (0.97,1.46)	0.103	1.18 (0.93, 1.51)	0.179
Recessive	AA, AT	690 (90.0)	610 (88.4)	Ref	—	Ref	—
	TT	77 (10.0)	80 (11.6)	1.18 (0.84,1.64)	0.339	1.28 (0.87, 1.88)	0.208

Unadjusted (without covariates) and adjusted (for age, sex, BMI, hypertension and diabetes mellitus) multivariable logistic regression analysis was performed using different models (codominant, dominant, and recessive) between controls and ischemic stroke. OR: odds ratio; CI: confidence interval; Ref.: Reference group.

### Analysis of LTB4 levels

**Fig. 1:**
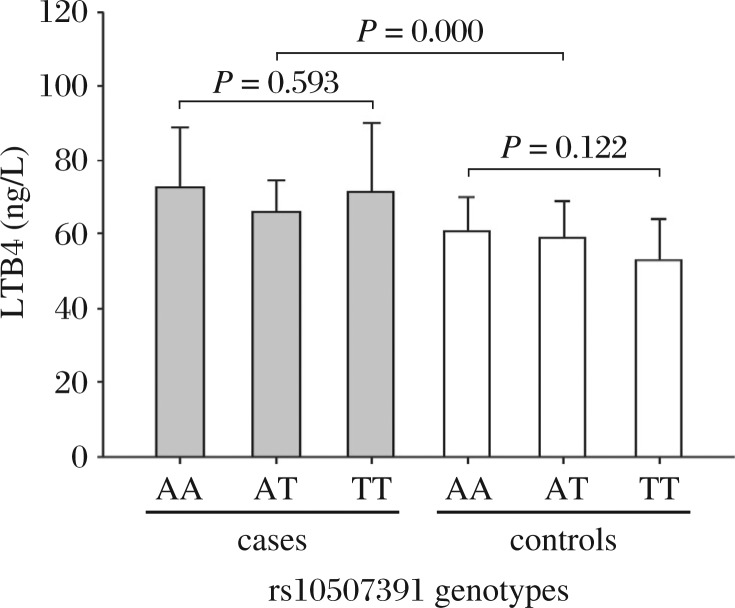
Box plot of the LTB4 levels in controls and ischemic stroke cases according to their rs10507391 genotypes. LTB4 levels are expressed in mean±SD. A significant difference in the mean levels of LTB4 could be observed between cases and controls with ischemic stroke cases showing higher levels than controls (70.06±14.75 *vs* 57.34±10.93 ng/L; *P* = 0.000). However, association between LTB4 levels and rs10507391 genotypes could be observed neither in the case group (*P* = 0.593) nor in the control group (*P* = 0.122).

To determine whether individuals with ischemic stroke had greater activity of the LT biosynthetic pathway than controls, the production of LTB4 (a key product of this pathway) was measured in plasma isolated from ischemic stroke cases and controls. LTB4 production analysis was conducted in 45 controls and 33 ischemic stroke cases (***Fig. 1***). A significant difference in the mean levels of LTB4 could be observed between cases and controls (*P* = 0.000) with ischemic stroke cases showing higher levels (70.06±14.75 ng/L) than controls (57.34±10.93 ng/L). However, association between LTB4 levels and the rs10507391 genotype could be observed neither in the case group (*P* = 0.593) nor in the control group (*P* = 0.122). Moreover, LTB4 levels did not differ between cases (72.57±16.22 ng/L, *n* = 9) and controls (60.89±9.73 ng/L, *n* = 12) carrying the AA genotype (*P* = 0.057). But a significant difference was found in the mean levels of T allele carriers between ischemic stroke cases (69.13±14.26 ng/L, *n* = 24) and controls (56.04±11.19 ng/L, *n* = 33, *P* = 0.000).

**Table 6:** Stratified analysis of the association between rs10507391 genotypes and ischemic stroke risk

Stratified characteristics	Genotype	Controls (*n*)	Cases (*n*)	*OR* (95%*CI*)	*P* value
Sex					
Male	AA	53	51	Ref.	—
	AT	186	198	1.11 (0.72, 1.71)	0.648
	TT	176	163	0.96 (0.62, 1.49)	0.865
Female	AA	47	46	Ref.	—
	AT	169	129	0.78 (0.49, 1.24)	0.296
	TT	136	103	0.77 (0.48, 1.25)	0.295
Age (years)					
≥60	AA	78	70	Ref.	—
	AT	269	271	1.12 (0.78, 1.62)	0.534
	TT	256	210	0.91 (0.63, 1.32)	0.635
< 60	AA	22	27	Ref.	—
	AT	86	56	0.53 (0.28, 1.02)	0.056
	TT	56	56	0.82 (0.42, 1.60)	0.551
BMI (kg/m^2^)					
≥24	AA	35	47	Ref.	—
	AT	127	170	1.00 (0.61, 1.63)	0.990
	TT	114	144	0.94 (0.57, 1.55)	0.811
< 24	AA	65	50	Ref.	—
	AT	228	157	0.90 (0.59, 1.36)	0.606
	TT	198	122	0.80 (0.52, 1.23)	0.314
Hypertension					
Yes	AA	26	73	Ref.	—
	AT	108	260	0.86 (0.52, 1.42)	0.547
	TT	77	199	0.92 (0.55, 1.55)	0.754
No	AA	74	24	Ref.	—
	AT	247	67	0.84 (0.49, 1.43)	0.511
	TT	235	67	0.88 (0.52, 1.50)	0.636
Diabetes					
Yes	AA	11	31	Ref.	—
	AT	45	105	0.83 (0.38, 1.79)	0.631
	TT	39	99	0.90 (0.41, 1.97)	0.793
No	AA	89	66	Ref	—
	AT	310	222	0.97 (0.67, 1.39)	0.850
	TT	273	167	0.83 (0.57, 1.20)	0.310

OR: odds ratio; CI: confidence interval; Ref.: Reference group.

**Table 7:** Haplotype analysis of ischemic stroke

Haplotype^a^	Controls		Cases		*OR* (95% *CI*)	*P*^b^
	2*N*	Frequency	2*N*	Frequency		
TA	972	0.6333	847	0.6133	Ref	—
AA	83	0.0544	62	0.0454	0.86 (0.61, 1.21)	0.376
AT	472	0.3074	459	0.3321	1.12 (0.95, 1.31)	0.174
TT	7	0.0049	12	0.0092	1.97 (0.77, 5.02)	0.149

^a^ The order of SNPs for inferring haplotypes was rs10507391, rs12429692 from left to right. ^b^ χ^2^ tests, comparison of the haplotype frequencies between controls and ischemic stroke. OR: odds ratio; CI: confidence interval; Ref.: Reference group.

## DISCUSSION

Since a genome-wide linkage analysis conducted by the deCODE group in an Icelandic population suggested that a four-SNP haplotype in the *ALOX5AP* gene conferred a nearly two times greater risk of stroke[7], several groups attempted to replicate the association of ischemic stroke with *ALOX5AP* variants. Genetic association studies in different populations were subsequently carried out. However, the results were controversial and conflicting. In particular, a significant association of ischemic stroke with *ALOX5AP* polymorphisms was found in Scottish[9], German[16], Chinese[14],[17], white American[18], Japanese[19], British[20], Spanish, and Portuguese[21] populations, whereas no evidence of a significant association was reported in different American[22]–[24], Swedish[25],[26], and Italian[27] cohorts.

The present study examined the association between variants of *ALOX5AP* and the risk of ischemic stroke in a Chinese Han population of eastern China. The investigated SNPs herein were selected on the basis of earlier reports, in which significant associations with ischemic stroke had been demonstrated. However, the results of this study suggested a lack of association between two SNPs in *ALOX5AP* and ischemic stroke risk. Several explanations are possible for such disparate results. There might be substantial genetic heterogeneity for ischemic stroke, leading to varied results in different study populations. Other population-specific genetic differences also might account for divergent results among ischemic stroke patients in different countries. Random chance might produce spurious positive associations in some populations and studies, but not in others[28]. To minimize the possible founder effect of some ethnic groups, further study is required to verify the validity of the association with new stroke population according to distinct ethnicity.

ALOX5AP participates in the initial steps of LT synthesis. Arachidonic acid is converted to LTA4 by the action of 5-LO and ALOX5AP. LTA4 is then metabolized either to proinflammatory LTB4 or to the vasoconstrictive and proinflammatory cysteinyl LTs[29].

The deCODE group detected that the amount of LTB4 synthesized by ionomycin-stimulated neutrophils from individuals with myocardial infarction was greater than that produced by those from control individuals. Furthermore, the observed difference in the release of LTB4 was largely accounted for by the fact that carriers of at-risk haplotype produce more LTB4 than non-carriers[7]. Although LTB4 production was not measured in cells from patients with ischemic stroke, a similar increase would be expected, given that at-risk haplotype of ALOX5AP showed similar association with myocardial infarction and ischemic stroke. Elevated levels of LTB4 might contribute to atherogenesis or plaque instability by promoting inflammation at atherosclerotic plaques, which supported the notion that increased activity of the LT pathway plays an important role in the development of myocardial infarction and ischemic stroke[30],[31].

In the present study, higher plasma LTB4 levels were observed in ischemic stroke cases than in controls, and LTB4 levels did not depend on the genotype of rs10507391 in cases or controls, respectively. This might indicate that during the acute phase of ischemic stroke, LTB4 levels are increased probably as part of the inflammation process. The lack of association with the genotype in cases and controls could be due to a general increase in LTB4 levels, masking the effect of the polymorphism. In addition, the fact that T allele carriers in cases presented statistically higher LTB4 levels than in controls suggested that T allele was associated with high LTB4 levels. However, it is not consistent with the association result of the rs10507391 allelic frequency with ischemic stroke. This could be explained by additional variants in *ALOX5AP* that have not been investigated, or in other genes belonging to the LT pathway, which may account for up-regulation of the LTB4 response. Further studies are needed to search for other potential causative variants in *ALOX5AP* and other genes involved in the LT pathway.

This study also presents several limitations. First, the proportion of males in cases was higher than in controls (59.7% *vs* 54.1%). To reduce a possible sex stratification effect, subgroup analysis stratified by sex was conducted. Moreover, TOAST subtype analysis of ischemic stroke cases showed non-significant association with genetic variants in *ALOX5AP*, although varied risk factors and phenotypic differences among ischemic stroke are mainly related to different stroke etiologies. Second, the sample size in the present study (690 cases and 767 controls) might not be large enough to detect a small effect of potential low-penetrance SNPs. Additionally, there was possible selection bias since the controls were partly recruited from hospital. Third, each single susceptible polymorphism might only contribute to a modest effect; thus analysis of a single SNP could be confused by unstudied SNPs that influence the phenotype. The combined effects of multiple variants of a gene or multiple genes would capture more information about ischemic stroke risk and provide a more comprehensive evaluation of genetic contribution to the risk of ischemic stroke. Therefore, more studies are needed to demonstrate the gene-gene interactions affecting the susceptibility to ischemic stroke.

In conclusion, the present study investigated the role of variants of *ALOX5AP* in the risk of developing ischemic stroke, and suggested no association between the two SNPs and ischemic stroke risk in a Chinese Han population of eastern China. Racial differences in the frequencies of genotypes and alleles may account partly for the different association findings between studies. Moreover, carrying T allele of the rs10507391 variant was associated with higher plasma LTB4 levels, which may result in a more evident proinflammatory activity and progression of atherosclerosis. Further rigorous genetic association studies, designed for the investigation of gene-gene and gene-environment interactions, might produce more conclusive results about the genetics of ischemic stroke.
